# Study on the distribution pattern of particle re-crushing in the coal and rock mass crushing process under pressure

**DOI:** 10.1371/journal.pone.0262235

**Published:** 2022-01-18

**Authors:** Shuang Song, Shugang Li, Tianjun Zhang, Hongyu Pan, Nan Liu, Lei Zhang

**Affiliations:** 1 College of Safety Science and Engineering, Xi’an University of Science and Technology, Xi’an, China; 2 College of Science, Xi’an University of Science and Technology, Xi’an, China; University of Sharjah, UNITED ARAB EMIRATES

## Abstract

In order to investigate the compaction and re-crushing characteristics of crushed coal gangue with different gradation, the compaction fractal test was carried out for crushed coal gangue with different Talbol power index n. The compaction deformation parameters such as displacement, stress-strain were analyzed according to the test results. And according to the test results, the compaction deformation relationship of the lateral limit crushed coal rock body considering the gradation is obtained. The test results show that: the crushing of coal rock particles exists in almost the whole compaction process of the crushed coal rock body, and the crushing of coal rock particles has a non-negligible influence on the deformation of the whole crushed rock body; the structural stability of large-size coal rock particles is not as good as that of small-size coal rock particles, and the large-size coal rock is more likely to be crushed under the same stress conditions; the distribution coefficient r of the crushed coal rock body decreases with the increase of axial stress; and before the Before the axial stress reaches 8 MPa, the distribution coefficient r of crushed rock samples tends to increase with n in general, and after reaching 8 MPa and later, the distribution coefficient r of crushed rock samples tends to decrease with n in general; the difference value of particle crushing increases with the increase of axial stress, and the weight value of particle crushing decreases with the increase of axial stress, and the changes of both are non-linear; according to the stress recovery in the compaction process of crushed coal rock body The compaction deformation model of the crushed coal rock body is constructed according to the crushing characteristics of coal rock particles in the stage of compaction, which effectively combines its fine action mechanism with macroscopic physical phenomena in a simple form and has certain practical engineering significance.

## 1 Introduction

In recent years, the fine view research of bulk filling materials has gradually received attention, and the compaction mechanical properties of bulk filling materials as the key factor of rock control in the coal mining technology of filling directly determine the effect and quality of filling mining [1, 2]. The grading structure of the crushed coal body is an important factor affecting the compaction and re-crushing characteristics of the crushed gangue, and the compaction mechanical properties of the crushed gangue directly determine the effect and quality of infill mining. The fine structure of bulk filling material determines its macroscopic mechanical properties, and the macroscopic damage behavior is also the result of the accumulation of rupture and damage behavior on the fine level [3, 4]. Therefore, the study of the distribution law of particle re-crushing during the pressure-bearing process of graded broken coal rock is of great significance for the safety management of the mining area and the prevention of surface subsidence.

In terms of rock particle fragmentation characteristics, Liu Hanlong et al. [5] obtained the hyperbolic equation of fragmentation rate with stress under triaxial test conditions for coarse-grained soils based on the fragmentation rate Br index proposed by Hardin [6]; the literature [7] studied the compression curve, creep behavior and the evolution law of particle fragmentation degree under dry and watery conditions by measuring the hardness of soil particles, Liu Mengcheng et al. [8] used the crushing rate proposed by Eniav [9] to define the peak crushing index; based on fractal theory, literature [10] investigated the fractal dimension of coarse-grained soils and the crushing degree of coarse-grained soils for the effects of coarse-grained content and coarse-grained soil water content on crushing rate, and concluded that the fractal dimension of coarse-grained soils increased with their water content under the same grading conditions. In terms of the intrinsic connection between compaction and re-crushing characteristics, the literature [11] conducted shear tests under different conditions of positive stress and relative density, and concluded that the response of specimens with a relative density of about 32% to shear showed a strain-softening pattern, and the specimens did not undergo a transition from shrinkage to expansion with increasing positive stress and super consolidation ratio; Kefen Zhang et al. [12] used numerical simulation of particle flow to studied the growth limit of fractal dimension during particle crushing; Sun Yifei et al. [13] used Eniav crushing index to establish a hyperbolic relationship between crushing index and shear strain; literature [14] conducted compression tests on sandy soil with various stress paths, and concluded that the plastic work calculated from the results of stress-strain relationship and relative crushing degree Br could be approximately linearized; Ding Jianyuan et al. [15] established logistic regression odds model after compaction of crushed particles to predict the growth trend of crushing degree.

The compressive strength characteristics of the filling material and the law of re-fragmentation are extremely important in the filling and mining technology. The above studies have done a lot of research on the compaction and crushing characteristics of crushed rock masses from macroscopic and microscopic perspectives, but there is less analysis on the intrinsic mechanism between compaction and re-crushing of graded crushed rock masses, and the mechanical intrinsic relationships in the compaction and crushing process need further depth. In this paper, the compression and fractal test of graded crushed gangue is based on fractal theory. The re-crushing characteristics of the graded crushed gangue in the compaction process are studied, and its fine action mechanism and macroscopic physical phenomena are effectively combined, so that the mechanical ontological model in the compaction process is established.

## 2 Methods

### 2.1 Test device and sample configuration

Based on the crushing mechanism of crushed coal and rock mass under pressure [16]. A self-designed and patented crushed rock mass compaction test system was employed to conduct compression tests of crushed coal gangue. This test system is a modern test system combining computer information collection and digital measurement instruments. The system can accurately measure the axial stress and axial displacement of rock samples during tests.

**Fig 1:**
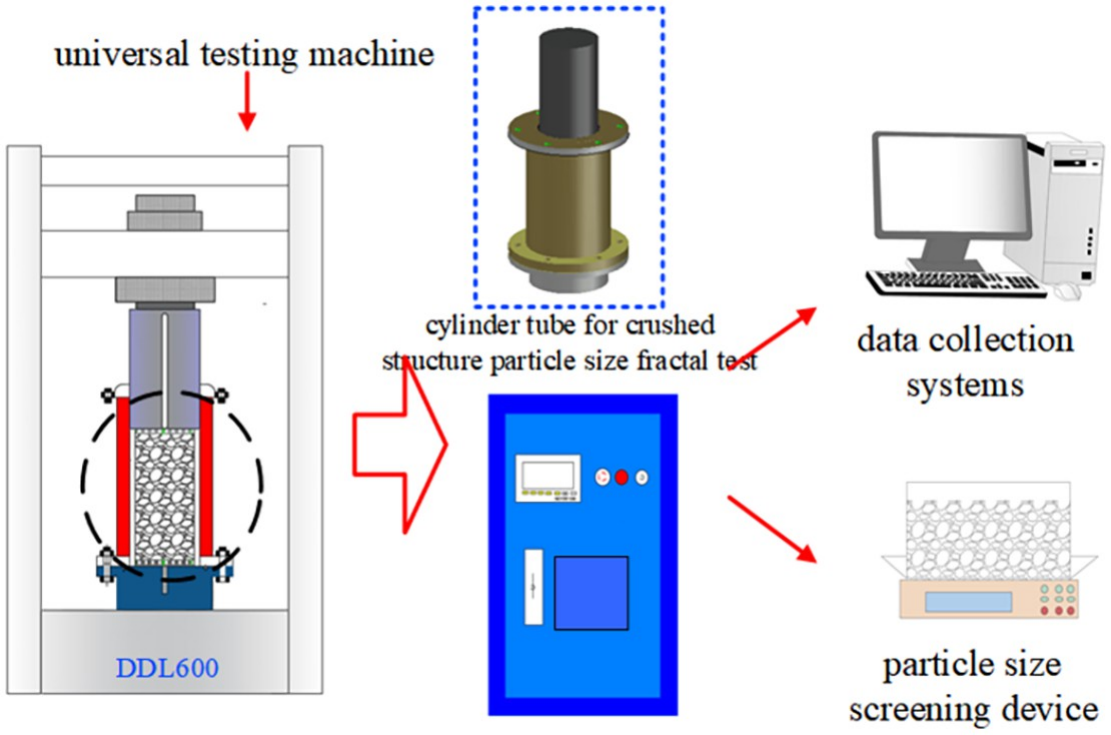
Test device.

The test system mainly includes a DDL600 electronic universal testing machine, crushed rock compression device, computer acquisition instrument, control system, vibration sorting screen and control cabinet. Among these components, the DDL600 electronic universal testing machine provides axial pressure for the cylinder under the setting and control of the computer control system. The computer controls the testing machine and collects the required data. The vibrating sorting screen device screens and separates particles into various particle size ranges in a controlled manner. The cabinet can display data changes such as the displacement and stress. In addition, tools such as steel rulers and electronic scales were required. The test device is shown in Fig 1.

**Fig 2:**
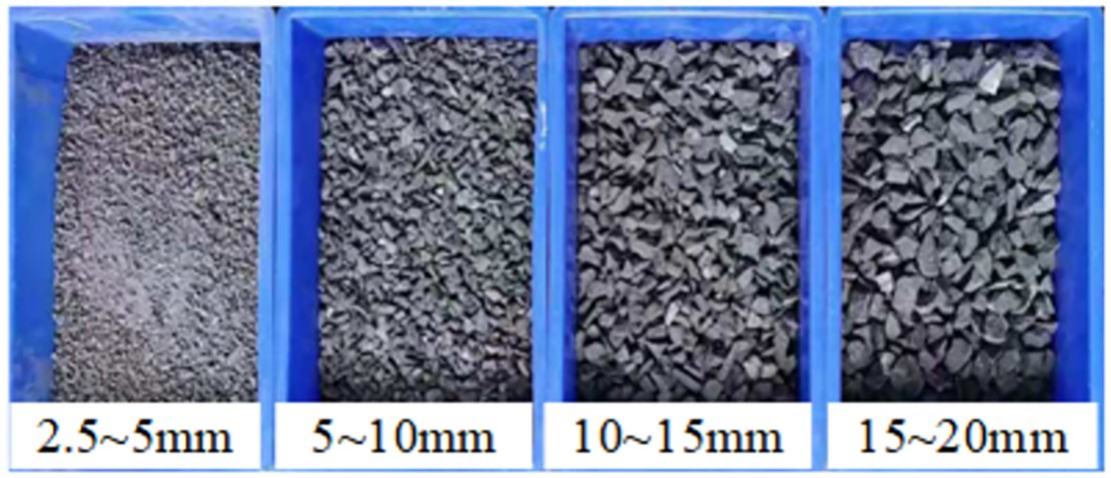
Coal gangue particles of various sizes.

The test raw materials were collected from the Cuijiagou Coal Mine in Shaanxi Province, and the density was measured as 1930 kg/m^3^. The raw materials were crushed in a crusher, and 2.5~5 mm, 5~10 mm, 10~15 mm and 15~20 mm particles were screened with the sorting screen, as shown in Fig 2.

According to the Talbol grading equation [17] (Eq (1)), the particle masses in all intervals (as listed in Table 1) with a total mass of 600 g were calculated for uniform mixing.

$$\frac{M_{\text{d}}}{M_{\text{t}}}={\left(\frac{d}{D}\right)}^{n}\times 100\text{\%}$$ (1)

where *d* is the particle size of the broken coal gangue, mm; *D* is the largest particle size of the broken gangue, mm; *M*_*d*_ is the mass of broken gangue in the rock sample with a particle size smaller than or equal to *d*, g; *M*_*t*_ is the total mass of broken gangue in the sample, g; and *n* is the Talbol power exponent.

**Table 1:** Sample mass distribution.

*n*	Different particle size distribution/g			
	2.5~5 mm	5~10 mm	10~15 mm	15~20 mm
0.2	196.5	174.5	122.1	107.5
0.4	179.1	169.3	135.4	116.2
0.6	165.3	158.9	143.4	133.4
0.8	160.9	151.6	148.2	139.3

### 2.2 Test procedure

**Fig 3:**
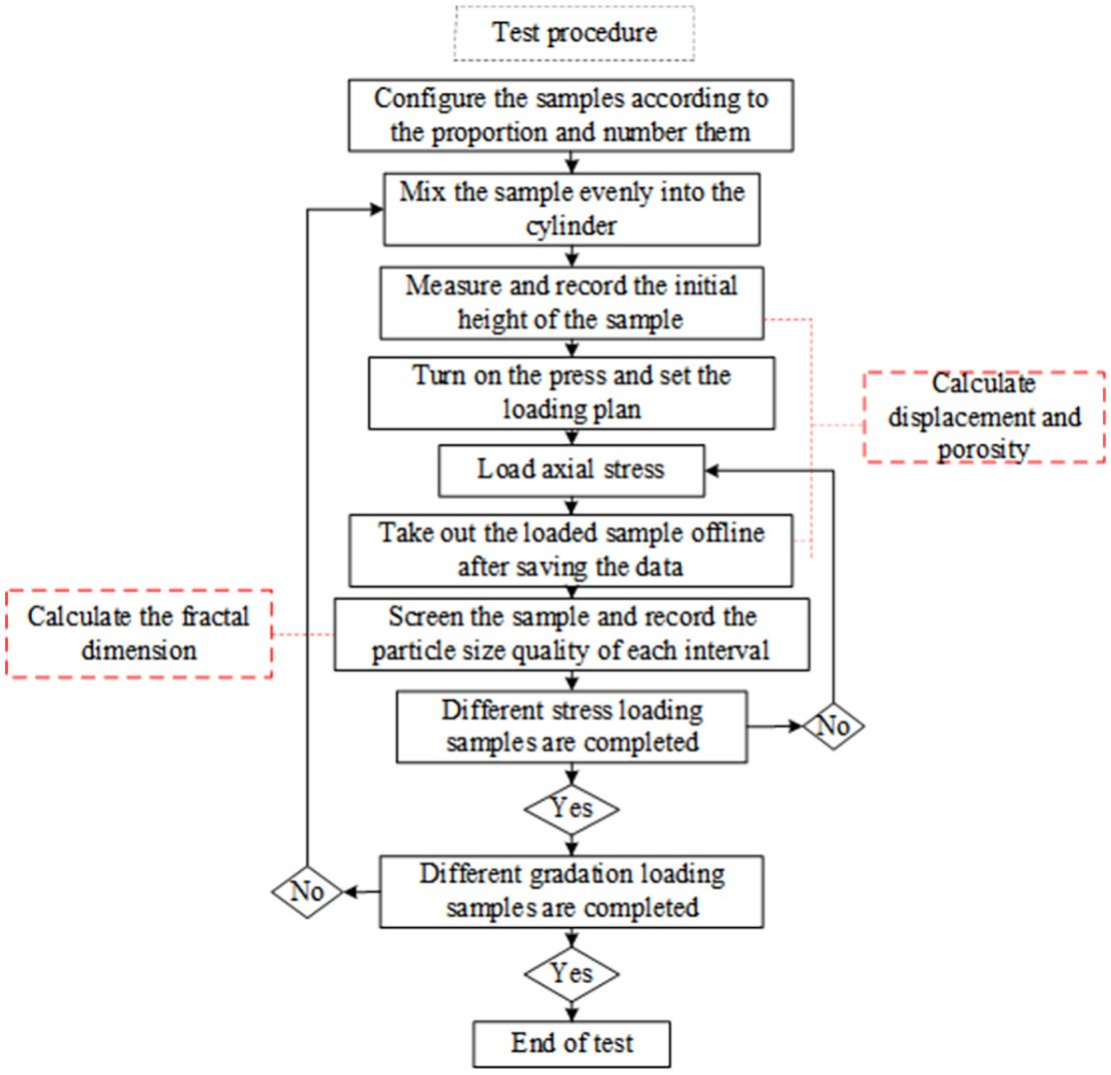
Test flow chart.

The above crushed rock mass compaction system with a DDL600 electronic universal testing machine as the loading device was implemented to determine the compaction characteristics of crushed coal gangue samples, and crushed coal gangue particles with the same gradation structure were loaded into the compression cylinder under confinement. The press was activated, and the target axial stress was applied to the broken coal and rock mass sample through the computer control system in a gradually increasing loading manner. When the load on the broken rock samples of the same grades reached the target stress value, the sample was maintained under the target stress for 30 minutes. This ensured that the broken coal and rock particles attained equilibrium, and the piston height changes after each axial stress loading step were recorded to calculate the sample porosity. Apply the next target stress until all target stress loads are completed, and then change to the next set of specimens. After sample measurement, stress and strain data were obtained through the computer data acquisition system and stored, the previous steps were repeated with other samples, and data were recorded until the test was completed. The test flow chart is shown in Fig 3.

### 2.3 Displacement changes in the crushed coal and rock mass samples under pressure

Pressure-bearing broken coal rock masses exhibit different mechanical properties and behaviours to those of complete rock masses in the compression process. In the literature [17], crushed coal rock mass samples were compacted according to the force characteristics of broken coal rock masses in the compression process. The process was divided into three stages: unconsolidated and broken stage, stress recovery stage and original rock stress stage. At the stress recovery stage, the crushed coal and rock mass samples were compacted and deformed under the action of confinement constraints and pressure, and a series of mechanical behaviours occurred, which gradually compacted the previously unconsolidated crushed coal and rock mass samples.

**Fig 4:**
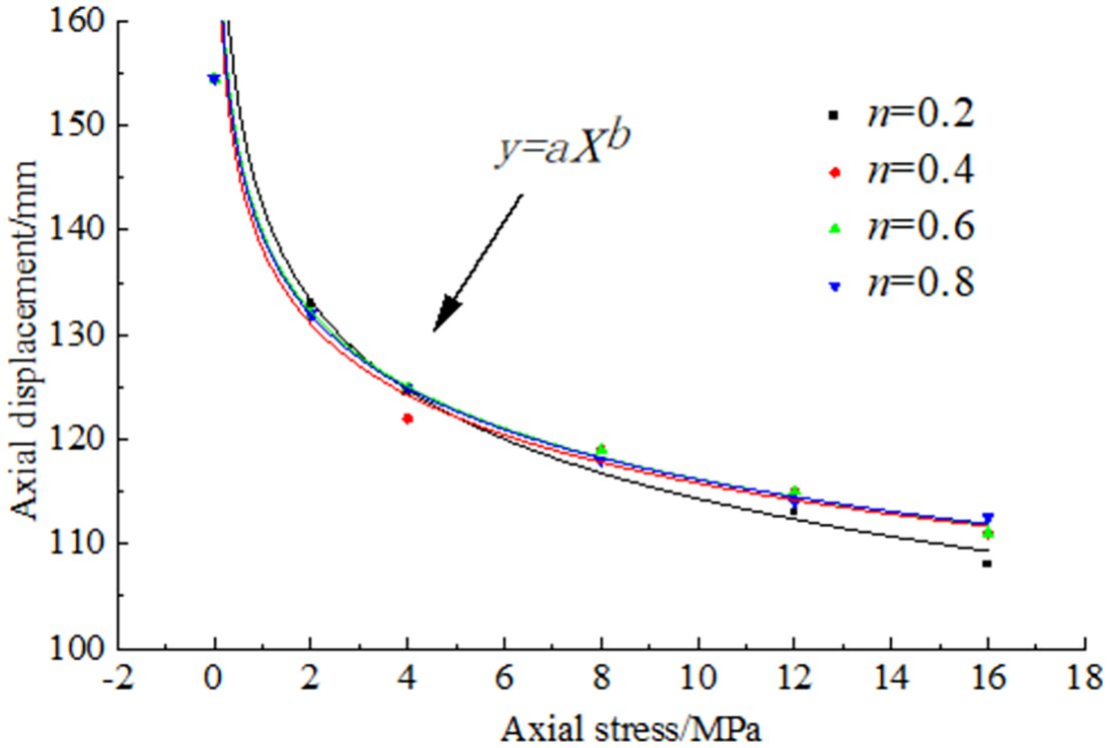
Relationship between the displacement and stress.

To study the mechanical properties of pressure-bearing broken coal and rock masses, the compaction changes in broken coal and rock masses under different Talbol power exponent values *n* and various axial stress levels were analysed. According to the test displacement data collected during the tests, a diagram of the change in the axial displacement with the axial stress was generated, as shown in Fig 4.

Fig 4 shows that the height of the broken coal and rock masses with the different Talbol power exponent values *n* exhibited an overall downward trend under the action of the various axial stress levels, but with increasing axial stress, the axial displacement rate decreased, and it was found that the relationship between the axial displacement and stress could be expressed with a power function. The sample axial displacement changed the fastest from 0~2 MPa, and all broken samples with different Talbol power exponent values *n* were reduced by more than 20 mm. At this point, the main deformation mechanism of the coal particles under stress was particle adjustment. After the stress reached 4 MPa, the filling process of the larger pores in the broken coal rock mass sample was basically completed due to particle filling. The process could no longer overcome the internal friction to reduce the porosity through inter-particle movement. At this point, under the higher axial stress, the coal and rock particles were increasingly broken and ground, which caused the large particles to decrease and fill any remaining voids. Therefore, the porosity of the broken coal and rock mass sample decreased, and the sample was further compacted when the inter-particle spaces were reduced to a certain extent. Under the action of a high stress, the broken coal rock mass sample experienced phenomena such as consolidation and bonding, which compacted the broken coal rock mass sample into a proto-like rock mass similar to the original rock mass.

### 2.4 Compaction model of broken coal and rock mass considering gradation

**Fig 5:**
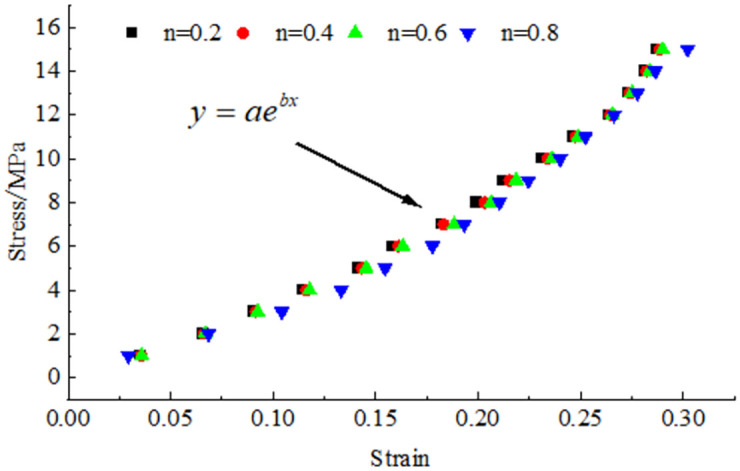
Stress-strain relationship of crushed coal rock mass with different grading structure.

To study the characteristics of the broken coal and rock masses with different Talbol power exponent values *n* in the compaction process, a stress–strain relationship diagram was generated, as shown in Fig 5, according to the stress–strain data collected during the experiments.

Ma *et al*. [18] proposed the following stress–strain function relationship for compacted and broken coal bodies through experimental analysis:

$$\sigma =a{\text{e}}^{b\varepsilon }$$ (2)

To determine the test parameters *a* and *b* in Eq (2), the data depicted in the stress–strain relationship diagram, as shown in Fig 5, were fitted to obtain a regression equation. The fitting relationship and related parameters are provided in Table 2.

**Table 2:** Stress–strain fitting relationship for the broken coal and rock masses under different values of *n*.

Sample	*n*	Fitting relationship	R^2^
G-1	0.2	*σ* = 1.659*e*^7.642*ε*^	0.988
G-2	0.4	*σ* = 1.612*e*^7.71*ε*^	0.989
G-3	0.6	*σ* = 1.566*e*^7.776*ε*^	0.991
G-4	0.8	*σ* = 1.493*e*^7.795*ε*^	0.991

The values of test parameters *a* and *b* in Eq (2) were obtained, as listed in Table 2. By drawing a curve based on fitting parameters *a* and *b* and Talbol index *n*, it was found that fitting parameters *a* and *b* could be described with exponential and linear functions, respectively, of the Talbol index *n*, as follows:

$$\begin{array}{l} a=a_{1}+a_{2}n\\ b=b_{1}+b_{2}b_{3}{}^{n} \end{array}$$ (3)

In the above equations, *a*_*1*_, *a*_*2*_, *b*_*1*_, *b*_*2*_, and *b*_*3*_ are the fitting parameters of the functional relationships. The fitting parameters and correlation coefficients of the functional relationships are summarized in Table 3.

**Table 3:** Compaction parameters and fitting parameters of *n*.

*a*		*b*			
*a*_1_	*a*_2_	*b*_1_	*b*_2_	*b*_3_	R^2^
3.088	-3.09	2.024	3.088	3.088	0.995

**Fig 6:**
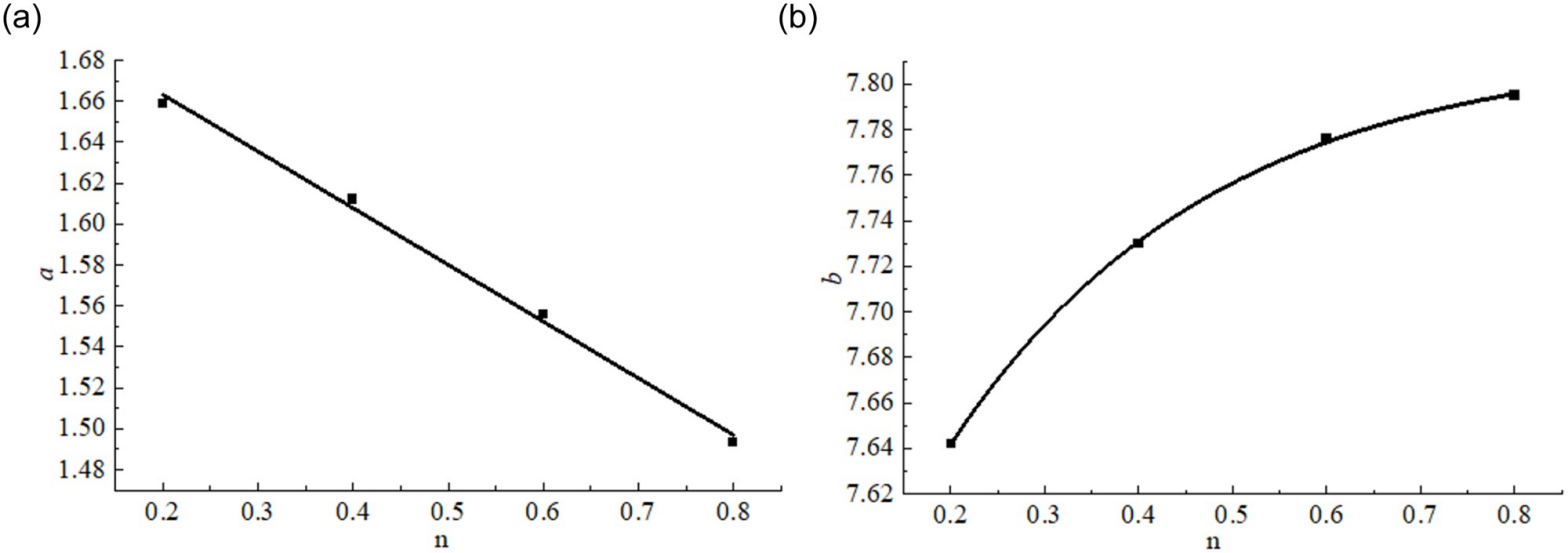
Relationship between the compaction parameters and *n*. (a) Relationship curve between *a* and *n*. (b) Relationship curve between *b* and *n*.

In summary, based on the compaction stress–strain relationship of the crushed coal and rock masses under pressure, the relationship between fitting parameters *a* and *b* and Talbol index *n* for the crushed coal rock mass samples was determined, as shown in Fig 6. The compaction constitutive model for the crushed coal and rock masses considering Talbol index *n* under confinement constraints is as follows:

$$\sigma =\left(a_{1}+a_{2}n\right)e^{\left(b_{1}+b_{2}b_{3}{}^{n}\right)\varepsilon }$$ (4)

where *a*_*1*_, *a*_*2*_, *b*_*1*_, *b*_*2*_, *b*_*3*_ and the other main parameters can be experimentally measured.

**Fig 7:**
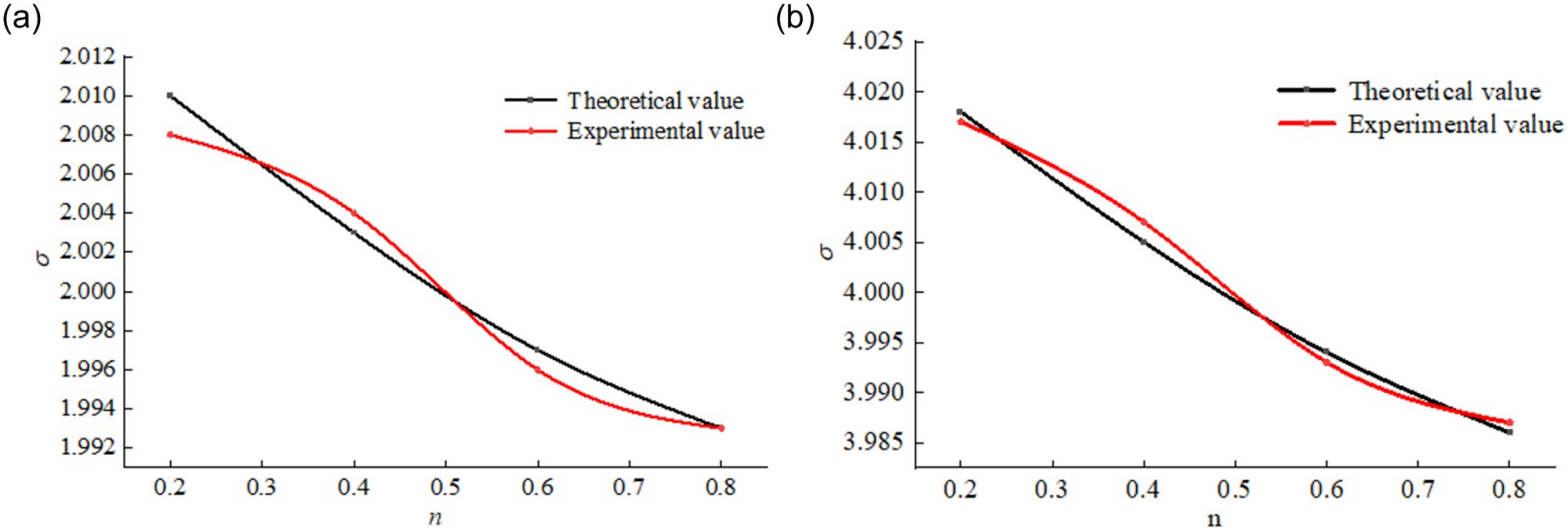
Comparison of the test and calculation results. (a) *ε* = 0.066. (b) *ε* = 0.116.

To verify the accuracy and rationality of the model expressed by Eq (4), combined with the stress and strain data measured in the tests, the stresses on the samples with the different gradation index values *n* under strains of 0.066 and 0.116 were obtained, and the test results were coupled with the constitutive relationship. A comparison chart of the calculation results is shown in Fig 7.

Fig 7 shows that the correlation between the theoretical and experimental curves of the stress change with *n* under the two different strain conditions was higher than 0.995 for the broken coal rock mass samples, and the correlation degree reached as high as 0.999 under a strain of 0.116. In the constitutive model for the compacted and broken coal mass samples considering the gradation structure, the calculation results were consistent with the test results, which verifies the rationality and accuracy of the proposed constitutive model.

## 3 Discussion

### 3.1 Characteristics of coal particle crushing

Broken coal and rock particles can deform under the action of axial stress, and the particle arrangement can accordingly vary. Coal and rock particles can move, rotate, break, crack, be ground, fill, and become consolidated under the action of contact forces. A series of complex effects results in a new particle arrangement balance. Each stress loading step can cause reorganization of broken coal and rock particles, thereby causing the entire broken coal and rock mass to undergo compaction deformation. The most intuitive manifestation in the experimental process entails the change in axial displacement.

**Fig 8:**
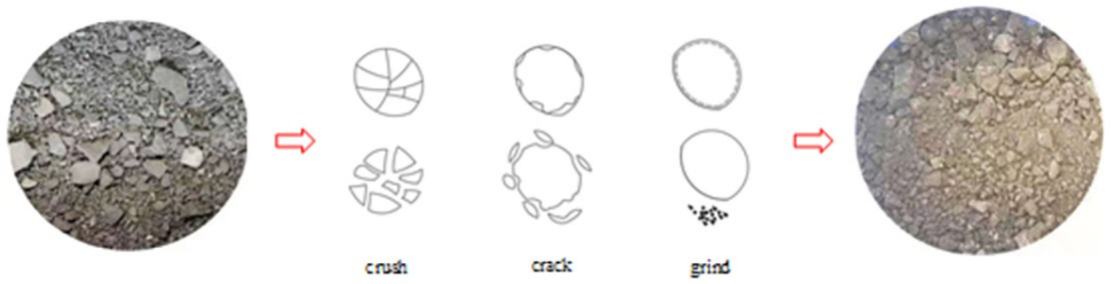
Coal particle crushing mode.

Fig 8 shows a physical comparison of a broken coal and rock mass sample before and after the test. The figure shows that the gradation structure of the broken coal and rock mass sample before the experiment is basically granular, the shape features are obvious, and the edges and corners are distinct. The broken particles are aligned along their axis during the experiment. A series of complex effects, such as crushing, cracking, and grinding, occurs under the action of axial stress, which causes large particles to become small particles or powder. After the test, the crushed coal particles are powdery, the prominent edges and corners of the particles have disappeared, and the shape is closer to a spherical shape.

In the establishment process of the confined compaction constitutive relationship in the study of the compaction stability features of crushed coal and rock mass samples under pressure, an exponential relationship could suitably represent the stress–strain relationship of the crushed coal and rock mass samples under pressure, and the fitting degree was very high. However, this relationship is only a mathematical expression and lacks physical meaning. In the analysis of the compaction process of the broken coal rock mass samples, it was found that the compaction and crushing process entails irreversible plastic deformation. Even if the final compaction degree were the same, the final rock mass differed from the original rock mass. Hence, the final rock mass could only be referred to as a proto-like rock mass. The essential reason for the irreversibility of the compaction and crushing process is energy consumption. The work achieved by external forces was consumed not only in the coal and rock particle crushing process but also in the particle filling and movement processes, mutual rubbing and mutual friction. Based on the above studies, it could be found that coal and rock particle crushing occurred throughout nearly the entire compaction process of the crushed coal and rock mass samples, and the crushing process of coal and rock particles imposed a non-negligible effect on the deformation of the entire crushed body.

In addition to the influence of external forces, the impact of the particle crushing degree mainly depended on the particle nature, such as the material hardness. The size of broken particles of the same material can differ, which can also affect the re-crushing state. The structural stability of large particles is typically not as high as that of small particles, and large particles may contain more defects. Compared to small particles under the same stress conditions, large particles are more readily broken.

### 3.2 Distribution coefficient of the graded coal and rock masses after re-fragmentation

To facilitate further analysis and study of the fractal pattern and mass distribution of the broken coal and rock masses with the different gradation structures under the various axial stresses, the measurement and calculation data obtained during the tests were compiled, as summarized in Table 4.

**Table 4:** Mass distribution.

Sample	Talbol index	P/MPa	Weight /g				
			0–2.5	2.5–5	5–10	10–15	15–20
G-1	0.2	2	151.57	98.09	166.81	100.13	83.4
		4	203.92	104.87	146.1	82.38	62.73
		8	264.78	102.87	127.6	66.55	38.2
		12	313.56	104.32	101.65	54.82	25.65
		16	370.85	81.58	83.54	46.75	17.28
G-2	0.4	2	128.48	85.85	184.45	115.35	85.87
		4	182.66	103.18	156.82	91.96	65.38
		8	243.18	107.91	119.93	80.92	48.06
		12	321.37	82.96	110.87	64.41	20.39
		16	372.55	84.01	88.6	45.28	9.56
G-3	0.6	2	123.99	88.74	184.03	116.99	86.25
		4	186.26	110.75	151.79	89.22	61.98
		8	257.49	108.21	130.93	64.46	38.91
		12	325.65	103.03	103.94	54.31	13.07
		16	377.93	90.93	84.35	34.31	12.48
G-4	0.8	2	115.13	87.21	185.96	119.9	91.8
		4	175.52	110.61	160.35	100.01	53.51
		8	262.15	111.63	128.37	68.5	29.35
		12	337.84	96.38	102.85	41.81	21.12
		16	389.4	87.5	77.42	28.54	17.14

Considering that crushed coal and rock masses can deform in the pressure-bearing process, large particles can experience cracking, crushing, grinding and other re-crushing effects, which can affect the original particle size distribution of the crushed particles. To analyse the changes in the particle size distribution of the graded broken coal and rock masses under the different axial stresses, the distribution coefficient *r* was introduced to describe the particle size distribution of the broken coal and rock particles [19]:

$$r=\sum_{n=1}^{k}W_{n}D_{n}$$ (5)

In the above equation, *k* is the number of rock sample groups in a given particle size interval; *D*_*n*_ is the maximum particle size in the n-th particle size interval, mm; and *W*_*n*_ is the percentage of the mass in the n-th particle size interval to the total mass of the rock sample, %. A smaller *r* value indicates that after rock sample deformation under loading, the proportion of small-size particles in the rock sample is greater.

According to the test results, the mass proportions in the broken coal and rock masses with the different Talbol power exponent values *n* were obtained, and the distribution coefficient *r* of the broken coal rock masses with the different Talbol power exponent values *n* could be determined by substituting the data into Eq (5). The distribution coefficient was calculated based on the Talbol power exponent *n*, as shown in Fig 8.

**Fig 9:**
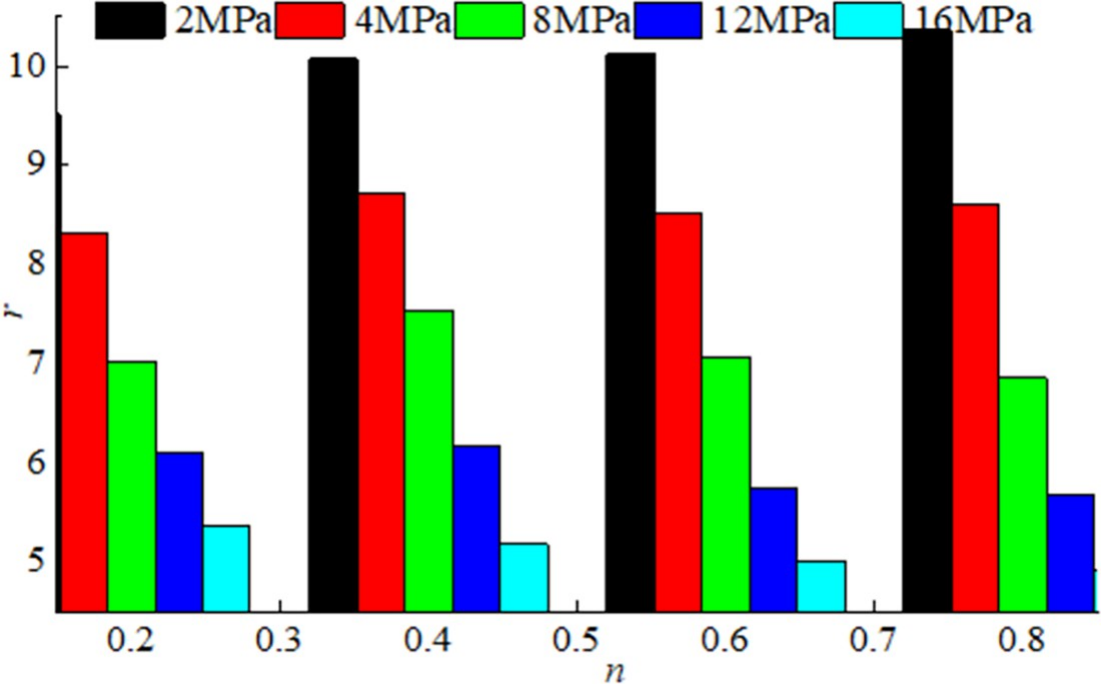
Histogram of *r*-*n*.

Fig 9 shows that the distribution coefficient *r* of the broken coal rock masses decreased with increasing axial stress before the axial stress reached 8 MPa. The distribution coefficient *r* of the broken rock samples exhibited an overall increasing trend with *n*. The distribution coefficient *r* of the fractured rock samples decreased up to 8 MPa and subsequently exhibited a decreasing trend with *n*. However, the distribution coefficient *r* of the broken coal and rock masses with the different Talbol power exponent values *n* fluctuated locally. In the loading process of the broken rock samples, large particles were continuously crushed, and small particles gradually increased. The particles in the broken rock samples became increasingly interlaced. The main skeleton was broken, producing more small particles, and the relationship between the distribution coefficient *r* and *n* values of the broken rock samples fluctuated.

### 3.3 Further discussion on the distribution coefficient under fragmentation

The distributions of the coal and rock particles in the samples with the different Talbol power exponent values *n* under the various axial pressures were analysed in great detail. Based on the test results, the percentage of 0~2.5 mm particles was defined as *p*_*1*_, the percentage of 2.5~5 mm particles was denoted as *p*_*2*_, the percentage of 5~10 mm particles was defined as *p*_*3*_, the percentage of 10~15 mm particles was denoted *p*_*4*_, and the percentage of 15~20 mm particles was defined as *p*_*5*_.

The ratio of *p*_*1*_ of the smallest particle size range, i.e., 0~2.5 mm, to *p*_*5*_ of the largest particle size range, i.e., 15~20 mm, was defined as the particle breakage difference *m*_*p*_. The ratio of the sum of *p*_*2*_, *p*_*3*_ and *p*_*4*_ to that of *p*_*1*_ and *p*_*5*_ was defined as the particle crushing weight *m*, and the evolution of the mean values of *m*_*p*_ and *m* was explored.

The calculation method of the mean values of *m*_*p*_ and *m* is as follows:

$$m_{p}=\frac{p_{1}}{p_{5}}$$

$$m=\frac{p_{2}+p_{3}+p_{4}}{p_{1}+p_{5}}$$ (6)

**Fig 10:**
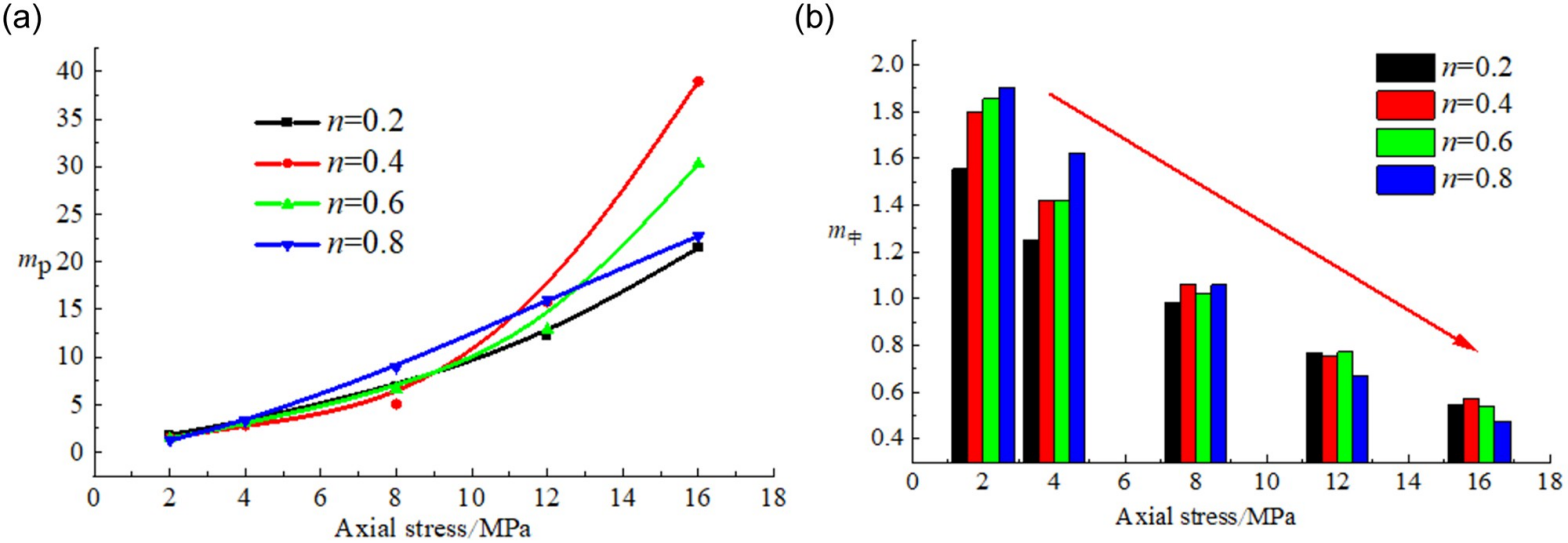
Variation in *m*_*p*_ and *m* with the axial stress. (a) Relationship between *m*_*p*_ and the axial stress. (b) Relationship between *m* and axial stress.

Fig 10 shows that the particle crushing difference *m*_*p*_ increased with increasing axial stress, and the particle crushing weight *m* decreased with increasing axial stress. The changes in both parameters occurred in a non-linear manner with the Talbol power exponent. Under an index value *n* of 0.8, the *m*_*p*_ value was larger than that under an *n* value of 0.2. In the process of gradually increasing the axial stress, the edges and corners of the large coal sample particles were broken under force application, resulting in a continuous increase in fine coal sample particles. Moreover, movement occurred to fill the spaces between the coal sample particles. Therefore, *m*_*p*_ increased with increasing axial stress. The particles of the broken coal sample with a Talbol power exponent of 0.8 were relatively large. Consequently, the phenomenon of large-particle coal fragmentation occurred more notable. Moreover, the *m*_*p*_ value was larger than that under an *n* value of 0.2. The change in the mean values of *m*_*p*_ and *m* could reflect the crushing degree of the mixed coal samples in the loading process to a certain extent.

### 3.4 Relationship between particle crushing and compaction deformation

In the compaction testing process of the crushed coal and rock masses under pressure, the particles of the broken samples with the different gradation structures could be regarded as spherical particles of varying sizes. The particles could be broken before they exhibited re-crushing behaviour and became more powdery particles. The compaction deformation process of each coal and rock mass sample as a whole was considered to be caused by the mesoscopic contact and movement phenomena of the particles. The mesoscopic mechanical analytical model proposed by Chang *et al*. [20, 21] was adopted to determine the stress–strain relationship for the macroscopic specimens by statistically averaging the mesoscopic displacement of the particle contact surfaces under loading.

In summary, in the establishment process of the constitutive compaction equation for the graded, crushed coal and rock mass samples, it was necessary to fully consider the mesoscopic compaction mechanism. In the study of the compaction process of the broken coal and rock mass samples, the compaction process was divided into three stages: plastic instability stage, stress recovery stage and proto-rock stress stage. According to the stress characteristics of the crushed coal and rock mass samples in the compression process, the deformation of the broken coal and rock mass samples at the stress recovery stage could be divided into two mechanisms: displacement reorganization and broken particle reorganization. Displacement reorganization occurred in two ways, namely, filling and sliding. At the stress recovery stage, the crushed coal and rock mass samples experienced compaction and deformation under the action of confinement constraints and pressure, and the bearing capacity was gradually recovered. The stress–strain relationship for the broken coal and rock mass samples at this stage can be expressed as [18]:

$$\sigma =a{\text{e}}^{b\varepsilon }$$ (7)

Therefore, in the construction process of the constitutive compaction equation for the graded crushed coal and rock mass samples, the strain [22, 23] ε was defined as the sum of the strains caused by particle filling, sliding and crushing, as follows:

$$\varepsilon =\varepsilon_{1}+\varepsilon_{2}+\varepsilon_{3}$$ (8)

where *ε* is the total axial strain, *ε*_*1*_ is the strain caused by particle filling, *ε*_*2*_ is the strain caused by particle sliding, and *ε*_*3*_ is the strain caused by particle breakage.

In the compaction and reorganization process of the graded broken coal particles, deformation was mainly attributed to the instability of the local pore structure between the broken coal particles. At the initial near-linear stage, the work achieved by the axial pressure was largely consumed to overcome the friction between the particles. The stress was concentrated near the pores. Once the stress near the pores exceeded the static friction force, local instability occurred near the pores. Subsequently, the particles overcame the internal friction and filled any gaps. Alternatively, inter-particle interlocking could occur. The deformation process at this stage could be approximated as a pre-sliding system. The deformation caused by local instability could be regarded as pre-slip displacement, which mainly includes plastic deformation and non-linear elastic deformation [24]. Since the deformation amount of the particles was very small, it was assumed that there occurred no irreversible plastic deformation between the particle contact points in this study.

Therefore, considering all graded broken coal and rock mass samples, a model with a damper and spring in parallel could be adopted to intuitively define a non-linear elastic model of the compaction deformation phenomena attributed to particle filling. The compaction caused by particle filling can be expressed as:

$$\varepsilon_{1}=\frac{\sigma_{1}}{E_{1}}(1-e^{E_{1}/\zeta_{1}})$$ (9)

In the above equation, *ζ*_*1*_ is the friction factor between the particles, *E*_*1*_ is the buckling deformation modulus, and *σ*_*1*_ is the axial stress.

At the particle sliding stage, the work achieved by the axial pressure was also mainly consumed to overcome the internal friction between the coal and rock particles, including static and sliding friction forces. According to the characteristics of the dynamic strength, the deformation process of the broken coal and rock mass samples could also be regarded as the dynamic process of the friction strength. In cohesive soils, the deformation process mostly includes the friction and bonding strengths [25]. As a frictional material, the strength of broken coal and rock masses does not include the bonding strength, but due to the unique particle shape, significant occlusion can occur between the particles [26, 27]. Therefore, the friction force overcome during deformation of the broken coal and rock masses can be divided into occlusal and sliding friction forces.

There exists a significant non-linear correlation between the internal friction angle of the samples and the plastic strain at the plastic deformation stage of the bulk particles [28, 29], and the form of the particles is characterized by an exponential function. During the compaction tests of the coal and rock masses, at the stress recovery stage, the compaction deformation reduction trend was highly obvious, and the samples exhibited significant non-linear characteristics. In this study, it was proposed to apply the Kelvin model to characterize the compaction deformation induced by particle sliding, and the corresponding equation is as follows:

$$\varepsilon_{2}=\frac{\sigma_{1}}{E_{2}}(1-e^{E_{2}/\zeta_{2}})$$ (10)

In the above equation, *ζ*_*2*_ is the coefficient of friction between the particles (including occlusal and sliding friction forces), and *E*_*2*_ is the sliding deformation modulus between the broken particles.

In regard to crushed coal and rock particles, under the action of axial loading, the crushing strength of the particles can reveal notable time-dependent characteristics [30]. The higher the external load, the more profound the deterioration in the particle strength is and the more notably the particles are broken. The shorter the time required, the higher the deformation rate of the entire sample is. Based on these considerations, a viscous pot element was applied in this study to characterize the deformation attributed to particle crushing. The equation is expressed as follows:

$$\varepsilon_{3}=\frac{\sigma_{1}}{\zeta_{3}}$$ (11)

In the above equation, *ζ*_*3*_ is the particle crushing viscosity coefficient.

**Fig 11:**
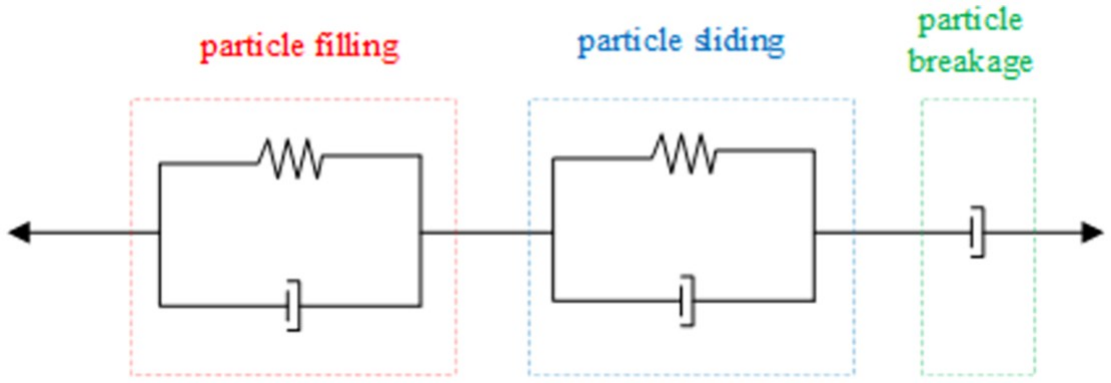
Composition of the compaction constitutive model.

Based on the above discussion, it could be considered that the crushed coal and rock mass compaction model comprised two Kelvin composite elements and one viscous pot element, as shown Fig 11. One Kelvin element was used to characterize the deformation caused by particle movement to fill pores in the compaction process, and the other Kelvin element could be employed to characterize the deformation due to particle sliding in the compaction process. A viscous pot element was applied to characterize the deformation attributed to particle crushing within the sample in the compaction process.

Although particle packing and particle sliding were both described with Kelvin components, the two Kelvin components exhibit different stages and methods. The particle packing phenomenon described with a Kelvin component mainly occurred at the initial near-linear stage, i.e., the compaction deformation rate was relatively low at the beginning. At the rapid stage, the other Kelvin component was mainly considered, and the compaction deformation rate decreased. In contrast, the viscous pot element mainly functioned at the compaction stage where the compaction deformation rate tended to stabilize. By connecting the two Kelvin components and viscous pot element in series to establish a compaction model for the broken coal and rock mass samples, the compaction equation can be expressed as:

$$\begin{array}{ll} \varepsilon & =\varepsilon_{1}\text{+}\varepsilon_{2}\text{+}\varepsilon_{3}=\frac{\sigma_{1}}{E_{1}}\left(1-e^{E_{1}/\zeta_{1}}\right)\text{+}\\ & \frac{\sigma_{1}}{E_{2}}\left(1-e^{E_{2}/\zeta_{2}}\right)\text{+}\frac{\sigma_{1}}{\zeta_{3}} \end{array}$$ (12)

This equation can effectively reflect the three-stage characteristics of the compaction and deformation process of broken coal and rock masses. This model effectively combines mesoscopic mechanical and macro-physical phenomena. The proposed equation is simple in form and provides a certain practical engineering significance.

## 4 Conclusion

1. After the stress reaches 4 MPa, the filling process of the large pores in the broken coal rock mass samples is basically completed under particle filling. When the inter-particle pores are reduced to a certain extent, the broken coal rock mass experiences consolidation and bonding under the action of high stress, which further compacts the broken coal rock mass into a proto-like rock mass very similar to the original rock mass. Coal and rock particle crushing occurs throughout nearly the entire compaction process of the crushed coal and rock mass samples, and coal and rock particle crushing imposes a non-negligible effect on the deformation process of the entire crushed body.
2. A relationship between the Talbol power exponent *n* and the various parameters of the stress–strain relationship for the crushed coal and rock mass samples under confinement constraints is obtained through interpolation. A constitutive compaction relationship for the crushed coal samples is established considering gradation. Under the same pressure conditions, the larger the *n* of the broken coal and rock mass, the higher the strain rate, and the larger the *n*, the easier it is to compact the broken coal rock mass. The test data are substituted into this constitutive relationship, which verifies its accuracy and rationality.
3. The distribution coefficient *r* of the broken rock masses decreases with increasing axial stress. Before the axial stress reaches 8 MPa, the distribution coefficient *r* of the broken rock samples generally increases with *n*. However, when the axial stress exceeds 8 MPa, the distribution coefficient *r* of the rock samples tends to decrease with *n* in general. The particle breakage difference increases with increasing axial stress, while the particle breakage weight decreases with increasing axial stress. Both changes are non-linear changes. When the Talbol power index n is 0.8, the *m*_*p*_ value is greater than that when n = 0.2, and the change rate of the mean *m* value is higher than that when n = 0.2
4. According to the crushing characteristics of the coal particles at the stress recovery stage during the compaction of the crushed coal and rock mass samples, two Kelvin components are connected in series to establish a compaction model for the crushed coal and rock mass samples. This model can effectively reflect the three-stage characteristics of the compaction and deformation process of the broken coal and rock mass samples, and the model effectively combines mesoscopic mechanical and macro-physical phenomena. The proposed model is simple in form and provides a certain practical engineering significance.

## Supporting information

S1 File The displacement and stress variation data, mass distribution, and the relationship between m and axial stress. (DOCX)
