# Supplementary material for: Study on the distribution pattern of particle re-crushing in the coal and rock mass crushing process under pressure
Source: PLoS One. 2022 Jan 18;17(1):e0262235. doi: 10.1371/journal.pone.0262235 (PMC8765633; doi:10.1371/journal.pone.0262235)
Supplement: S1 File — (DOCX) [file pone.0262235.s001.docx]

Supporting Information

Table 1 shows the displacement and stress variation data collected in the experiment, corresponding to Figure 4

**Table 1 Displacement changes with stress**

| Axial stress | n=0.2 | n=0.4 | n=0.6 | n=0.8 |
| --- | --- | --- | --- | --- |
| 0 | 15.45 | 15.45 | 15.45 | 15.45 |
| 2 | 13.3 | 13.2 | 13.2 | 13.2 |
| 4 | 12.45 | 12.2 | 12.5 | 12.5 |
| 8 | 11.8 | 11.9 | 11.9 | 11.8 |
| 12 | 11.3 | 11.5 | 11.5 | 11.4 |
| 16 | 10.8 | 11.1 | 11.1 | 11.25 |

Table 2 shows the particle size and mass distribution data of crushed coal and rock mass after compaction has been given in the manscript

**Table 2 Mass distribution**

| Sample | Talbol index | P/MPa | Weight /g | | | | |
| --- | --- | --- | --- | --- | --- | --- | --- |
|  |  |  | 0-2.5 | 2.5-5 | 5-10 | 10-15 | 15-20 |
| G-1 | 0.2 | 2 | 151.57 | 98.09 | 166.81 | 100.13 | 83.4 |
|  |  | 4 | 203.92 | 104.87 | 146.1 | 82.38 | 62.73 |
|  |  | 8 | 264.78 | 102.87 | 127.6 | 66.55 | 38.2 |
|  |  | 12 | 313.56 | 104.32 | 101.65 | 54.82 | 25.65 |
|  |  | 16 | 370.85 | 81.58 | 83.54 | 46.75 | 17.28 |
| G-2 | 0.4 | 2 | 128.48 | 85.85 | 184.45 | 115.35 | 85.87 |
|  |  | 4 | 182.66 | 103.18 | 156.82 | 91.96 | 65.38 |
|  |  | 8 | 243.18 | 107.91 | 119.93 | 80.92 | 48.06 |
|  |  | 12 | 321.37 | 82.96 | 110.87 | 64.41 | 20.39 |
|  |  | 16 | 372.55 | 84.01 | 88.6 | 45.28 | 9.56 |
| G-3 | 0.6 | 2 | 123.99 | 88.74 | 184.03 | 116.99 | 86.25 |
|  |  | 4 | 186.26 | 110.75 | 151.79 | 89.22 | 61.98 |
|  |  | 8 | 257.49 | 108.21 | 130.93 | 64.46 | 38.91 |
|  |  | 12 | 325.65 | 103.03 | 103.94 | 54.31 | 13.07 |
|  |  | 16 | 377.93 | 90.93 | 84.35 | 34.31 | 12.48 |
| G-4 | 0.8 | 2 | 115.13 | 87.21 | 185.96 | 119.9 | 91.8 |
|  |  | 4 | 175.52 | 110.61 | 160.35 | 100.01 | 53.51 |
|  |  | 8 | 262.15 | 111.63 | 128.37 | 68.5 | 29.35 |
|  |  | 12 | 337.84 | 96.38 | 102.85 | 41.81 | 21.12 |
|  |  | 16 | 389.4 | 87.5 | 77.42 | 28.54 | 17.14 |

Table 3 shows the relationship between m and axial stress

**Table 3 Relationship between *m* and axial stress**

| Axial stress | n=0.2 | n=0.4 | n=0.6 | n=0.8 |
| --- | --- | --- | --- | --- |
| 2 | 1.55352 | 1.79916 | 1.85388 | 1.89953 |
| 4 | 1.25014 | 1.41896 | 1.41702 | 1.61974 |
| 8 | 0.98033 | 1.06016 | 1.02429 | 1.05832 |
| 12 | 0.76882 | 0.75562 | 0.77137 | 0.6715 |
| 16 | 0.54587 | 0.57023 | 0.53685 | 0.47587 |

Table 4 shows the relationship between mp and axial stress

**Table 4** **Relationship between *m_p_* and axial stress**

| Axial stress | n=0.2 | n=0.4 | n=0.6 | n=0.8 |
| --- | --- | --- | --- | --- |
| 2 | 1.81739 | 1.49622 | 1.43757 | 1.25414 |
| 4 | 3.25076 | 2.79382 | 3.00516 | 3.28013 |
| 8 | 6.93141 | 5.05993 | 6.61758 | 8.93186 |
| 12 | 12.22456 | 15.76116 | 12.91584 | 15.99621 |
| 16 | 21.46123 | 38.96967 | 30.28285 | 22.71879 |

The above is part of the data in this manuscript. There is also some data related to stress and strain related to other related research, which is temporarily unavailable.
